# The Role of Rating Valence in AI Skin Cancer App Acceptance: Eye-Tracking and Questionnaire Study

**DOI:** 10.2196/93489

**Published:** 2026-06-11

**Authors:** Inga Jagemann, Sabrina Hegner, Gerrit Hirschfeld

**Affiliations:** 1 School of Business, University of Applied Sciences and Arts Bielefeld, Interaktion 1, Bielefeld, 33619, Germany, 49 521106 70508; 2 School of International Business, City University of Applied Sciences Bremen, Bremen, Germany

**Keywords:** eye-tracking, mobile health, mHealth, artificial intelligence, AI, technology acceptance model, TAM, skin cancer screening

## Abstract

**Background:**

Artificial intelligence–based skin cancer screening apps (AISCSAs) offer diagnostic potential but face limited adoption. App store cues, such as ratings, may influence acceptance; yet, little is known about how users cognitively process app store information in high-stakes health contexts. To address this gap, eye-tracking was used to measure visual attention while participants evaluated a mock AISCSA app store listing.

**Objective:**

This study aimed to test whether a single negative rating captures visual attention and whether an extended technology acceptance model (TAM) can predict behavioral intention to use (BI) AISCSAs.

**Methods:**

Participants (N=76) evaluated a mock app store listing for an AISCSA under positive (n=42) or negative (n=34) rating conditions while their eye movements were recorded. Analyses combined fixation durations in defined areas of interest (AOIs) with self-reported measures of perceived usefulness (PU), perceived ease of use (PEOU), trust, BI, willingness to pay, and the self-rated importance of app attributes.

**Results:**

Normalized fixation durations (seconds per square pixel) revealed the highest attention to the description (0.166 s/px^2^), followed by the reviews (0.11 s/px^2^) and the ratings (0.04 s/px^2^), while the price and the data protection received the least attention. Of the 5 self-rated app attributes, only reviews correlated positively with fixation durations on the reviews-AOI (*r*=0.28; *P*=.01). Rating valence had no significant effect on gaze patterns, PU, PEOU, trust, BI, or willingness to pay (all *P*s>.05). However, PEOU (*P*=.001), PU (*P*<.001), and trust (*P*<.001) were significantly correlated with BI.

**Conclusions:**

Although the expected attentional capture effect of the negative rating was not observed, the weak or nonexistent associations between fixation durations on the AOIs and the self-rated importance of app attributes suggest that eye-tracking captures aspects of information processing that are not directly reflected in self-reported evaluations. These findings indicate that eye-tracking provides a more direct approximation of actual user behavior by revealing implicit attentional processes beyond what is captured by questionnaires. While the technology acceptance model constructs and trust predicted BI, rating valence alone did not affect acceptance or gaze behavior. In high-stakes health contexts, textual information may outweigh rating valence in driving adoption. Future research should explore conditions under which rating valence matters, including more extreme rating contrasts, variations in accompanying review texts, and the influence of individual differences such as preexisting attitudes toward artificial intelligence and levels of artificial intelligence literacy.

## Introduction

### Background

Skin cancer is a highly prevalent global health concern, with melanoma, the deadliest subtype, posing a significant challenge. Early detection is crucial for improving survival rates, and clinical guidelines recommend full-body skin examinations by trained dermatologists and the use of dermoscopy for assessing skin lesions [1,2]. However, a growing shortage of dermatologists is exacerbating diagnostic delays [3,4]. Even today, more than half of all skin cancers are first noticed by patients themselves rather than detected during professional screenings [5], which has driven the exploration of artificial intelligence (AI) to support skin cancer detection. Empirical studies show that deep learning models can achieve diagnostic performance comparable to human experts in analyzing dermoscopic images for melanoma [6,7]. When integrated into mobile apps, these systems provide immediate risk assessments through automated, deep learning–based image analysis. Despite their potential to support early detection, adoption rates remain low, as many users continue to prefer conventional dermatological consultations [8-10].

This reluctance reflects a broader adoption gap identified in research on AI in health care—a discrepancy between the high diagnostic performance of AI systems and their limited acceptance in real-world practice [11,12]. While this gap is well-documented, less is known about the specific digital cues that drive such skepticism. Given that users increasingly rely on app store information to evaluate AI-based health apps, understanding how user-generated ratings shape acceptance becomes critical—particularly as these ratings represent a powerful yet understudied influence on decision-making in high-stakes health care scenarios.

Despite extensive research on rating valence in e-commerce, 3 key gaps remain. First, the role of rating valence in high-stakes health contexts, such as AI–based skin cancer screening apps (AISCSAs), remains largely unexplored. Second, prior research has not systematically integrated rating valence into the technology acceptance model (TAM) [13], particularly with regard to its causal effects on perceived usefulness (PU), perceived ease of use (PEOU), trust, and outcomes such as behavioral intention to use (BI) and willingness to pay (WTP). Existing studies predominantly rely on observational, nonexperimental designs, limiting causal inference. In contrast, this study uses an experimental manipulation of rating valence (positive vs negative) to directly examine its causal impact on these constructs. Third, prior work relies almost exclusively on self-reported measures and thus provides limited insight into the underlying cognitive processes, neglecting objective behavioral data such as eye-tracking.

To address these gaps, this study develops an integrative framework that combines the TAM, trust, and rating valence within a dual-process perspective of technology acceptance. By linking experimentally manipulated rating valence with both self-reported measures and eye-tracking data, we provide novel insights into how rating valence causally shapes acceptance beliefs and intentions, how users visually process app store information, and how closely self-reported evaluations align with actual attentional behavior.

### Theoretical Foundation and Research Hypotheses

#### Effects of Rating Valence on Technology Acceptance

Traditional technology acceptance research, particularly the TAM, assumes that technology evaluation is primarily a deliberate and analytical process. The TAM posits that PU and PEOU are the key determinants of BI [13]. However, dual-process theory suggests that human cognition is governed by 2 interacting systems: type 1 processing, which is fast, intuitive, and heuristic-based, and type 2 processing, which is slower, reflective, and cognitively effortful [14]. Because individuals tend to act as “cognitive misers,” they typically default to type 1 processing and rely on heuristic cues, particularly in uncertain or information-rich environments [14].

In digital marketplaces, such as app stores, these heuristic processes are highly relevant. User-generated star ratings serve as salient cues that shape users’ initial impressions before more deliberate evaluations take place. In this context, ratings and reviews function as key signals of product reliability by conveying aggregated user experiences and recommendations [15-17]. Meta-analytic evidence confirms that review valence exerts a powerful effect on purchase intentions [18]. While positive reviews enhance perceived quality and download likelihood [19-21], negative reviews often exert even stronger effects because they are perceived as more diagnostic and trustworthy [22,23]. This asymmetry is attributed to the negativity bias, where negative information attracts greater attention and has a stronger impact on decisions than positive ones [24-26].

Taken together, this suggests that rating valence may act as an early heuristic signal that shapes subsequent evaluations before more analytical processing occurs. Acceptance beliefs, such as PU, PEOU, trust, BI, and WTP, may already be biased by initial exposure to rating information. Importantly, WTP appears less stable than BI and may be influenced in a more complex and indirect manner, as it reflects distinct evaluative judgments and is shaped by both functional and psychological factors [27-29]. Building on this reasoning, this study introduces an experimental manipulation of rating valence, enabling a direct test of how external heuristic cues causally influence the formation of PU, PEOU, trust, and outcomes such as BI and WTP. Accordingly, we address the following research question: What is the effect of positive versus negative ratings on behavioral intentions? To empirically examine this proposed preprocessing role of rating valence across different outcome variables, the following hypothesis is formulated:

- H1: Rating valence significantly influences user perceptions and intentions, such that a negative rating leads to lower (a) trust, (b) PU, (c) PEOU, (d) BI, and (e) WTP.

#### Extending the TAM

**Figure 1.**
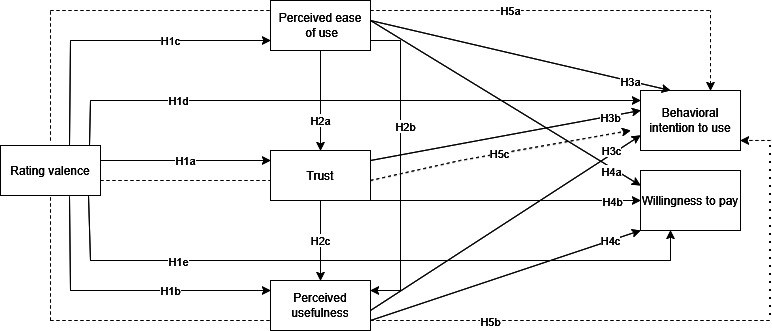
Integrative research model incorporating rating valence and trust into the technology acceptance model to predict behavioral intention to use and willingness to pay. H: hypothesis.

While the previous hypothesis proposed that rating valence influences initial evaluations, it remains unclear through which mechanisms these effects translate into acceptance decisions. The TAM provides a well-established framework to explain how user beliefs shape behavioral intentions. However, in AI-based health care contexts, the TAM alone may not fully capture user acceptance. A recent review by Lee et al [30] shows that the core TAM constructs are insufficient in complex health care environments, where additional factors, such as trust, organizational support, and education, significantly influence adoption. Due to the complexity, opacity, and perceived unpredictability of intelligent systems, trust emerges as an essential additional construct. Trust reflects the belief that a system performs reliably and safely, thereby reducing perceived risk and supporting decision-making under uncertainty [31,32]. Empirical evidence indicates that trust not only directly affects BI [22,33,34] but also positively influences PU [35-38]. Based on this framework, the following hypotheses are proposed (Figure 1):

- H2: Within the AISCSA context, PEOU is positively associated with (a) trust and (b) PU, and (c) trust is positively associated with PU.
- H3: BI is positively associated with (a) PEOU, (b) trust, and (c) PU.
- H4: WTP is positively associated with (a) PEOU, (b) trust, and (c) PU.

We want to test whether the TAM is able to explain the effects of the experimental manipulation. Prior research extending the TAM in AI-based health care contexts predominantly relies on cross-sectional survey designs to examine relationships between the TAM constructs and additional factors (eg, trust and privacy concerns) without experimentally manipulating external inputs [33,34,39,40]. While these approaches allow for the estimation of theoretically grounded causal pathways, they are based on observational (nonexperimental) data and therefore remain limited in establishing causal effects. Against this background, a key question is whether the TAM can adequately explain how externally manipulated rating information translates into behavioral intentions. Accordingly, we address the following research question: To what extent can the TAM explain the effect of rating valence on behavioral intentions? Building on this reasoning, we propose the following hypotheses:

- H5: The effect of rating valence on BI is mediated by (a) PEOU, (b) PU, and (c) trust.

#### Visual Attention and the Processing of Heuristic Cues

While heuristic cues such as rating valence are assumed to influence evaluations, this assumption does not account for how users cognitively process such information at the moment of exposure. Traditional technology acceptance research has largely relied on self-reported measures to assess acceptance beliefs, thereby conceptualizing decision-making as a consciously accessible and predominantly rational process [13]. However, such measures are limited, as individuals are often unable to accurately report the processes underlying their decisions due to recall bias and introspective inaccessibility [41-43]. Eye-tracking offers an important window into these processes by capturing objective and continuous measures of visual attention and cognitive engagement. According to the eye-mind hypothesis, individuals process what they fixate on, making visual fixation a reliable indicator of ongoing cognitive processing [44,45]. In this context, fixation duration reflects cognitive effort and information processing depth, with longer fixations indicating more intensive processing [46]. From a dual-process perspective, fixation patterns can be interpreted as indicators of different processing modes: shorter fixations are associated with intuitive, heuristic-based (type 1) processing, whereas longer fixations suggest more deliberate and analytical (type 2) processing [47,48]. Importantly, eye-tracking captures nonconscious attentional processes and thus provides an unobtrusive and behaviorally grounded measure of information processing that complements self-reported evaluations. Here, we use eye-tracking to improve our understanding in 3 ways.

First, we investigate where people look for information in the app store when browsing for health-related apps. In digital environments, attention is inherently selective and shaped by both stimulus characteristics and user goals. Prior eye-tracking research suggests that users tend to focus on visually salient elements such as images [49-53]. However, app store-specific evidence indicates that decision-making often relies more heavily on textual information, particularly descriptions and user reviews [54]. At the same time, existing findings point to substantial variability in attention patterns. Eye-tracking studies show that while some users engage extensively with review content, others allocate more attention to product selection, with differences observed across user groups such as different age cohorts [55]. Conversely, privacy- and security-related information typically receives limited attention, especially when presented in less salient formats [56]. Users may overlook privacy policies despite expressing concerns and rely on heuristic cues when apps are perceived as necessary [57,58]. Taken together, these findings suggest that attention allocation in app store environments is neither uniform nor fully explained by existing theory. In particular, it remains unclear which types of information users prioritize when evaluating high-risk health apps. To address this gap, we descriptively analyze gaze patterns.

Second, we test whether users’ consciously reported importance of app attributes corresponds to their actual allocation of visual attention during decision-making. While self-reports and eye-tracking capture different levels of cognitive processing [59], they are expected to converge on attributes that users deem personally relevant. If an attribute is integrated into an individual’s conscious value system, it should actively guide attentional resources toward related information. Accordingly, we address the following research question: To what extent are the self-rated importance of app attributes reflected in users’ gaze patterns? Based on this reasoning, the following hypothesis is proposed:

- H6: There is a positive relationship between the self-rated importance of app attributes and actual visual attention (fixation durations) to the corresponding areas of interest (AOIs).

Third, we test the effect of our experimental manipulation on visual attention. Consistent with the negativity bias [24-26], prior eye-tracking research shows that negative information attracts greater visual attention and elicits more intensive cognitive processing [60,61]. Such heightened attention may reflect a shift from intuitive, automatic processing (type 1) to more deliberate and analytical processing (type 2), particularly when users encounter risky or conflicting information [47]. Supporting this view, evidence suggests that negative or misleading stimuli can activate type 2 processing and prompt deeper evaluation compared to consensus-based stimuli [62]. Based on this reasoning, the following hypothesis is proposed:

- H7: A single negative rating increases visual attention to user reviews, as reflected in longer fixation durations on the reviews-AOI.

#### Moderating Role of Attention and Individual Differences

While rating valence shapes perceptions through the TAM-related mechanisms, its impact is unlikely to be uniform across users and situations. Dual-process theory suggests that the influence of heuristic cues depends on processing depth: information that is processed more extensively exerts a stronger effect on subsequent judgments [63]. Visual attention provides an observable indicator of this processing depth, as information receiving more attention is more likely to be incorporated into decision-making [64]. Beyond mere information acquisition, research indicates that visual attention actively biases decisions through a causal amplification effect. According to this mechanism, fixations are not neutral; rather, they amplify the relative value signal of the attended information. Consequently, longer fixations increase the weighting of a specific cue, such as rating valence, within the value integration process, thereby exerting a disproportionate influence on the final evaluation [65,66]. This implies that rating valence should have a stronger impact on user beliefs and intentions when it receives greater visual attention. In other words, the effect of rating valence is not fixed but depends on how deeply the cue is processed and integrated into the decision-making process. To capture this conditional effect, we propose that visual attention moderates the relationship between rating valence and acceptance-related outcomes:

- H8: The effect of rating valence on (a) PU, (b) PEOU, (c) trust, and (d) BI is moderated by visual attention, such that higher fixation durations on the ratings-AOI strengthen this relationship.

Prior research suggests that individual differences influence how users process and respond to app store information. Factors such as age, sex, education, prior experience with health apps, and self-reported AI knowledge may shape reliance on heuristic cues as well as attention allocation and acceptance judgments [67-69]. Less experienced users, for instance, may rely more strongly on ratings, whereas more knowledgeable users may engage in more analytic processing. However, existing evidence does not support a comprehensive set of directional hypotheses for each characteristic. Therefore, these variables are included as control and exploratory factors to provide additional insights into users’ attention, perceptions, and behavioral intentions.

## Methods

### Participants and Recruitment

Participants were recruited via university mailing lists, flyers, and student WhatsApp groups. Eligibility criteria included a minimum age of 18 years and fluency in German. Data collection took place between April 28 and May 31, 2025. A total of 95 participants were initially recruited; 19 were excluded due to insufficient eye-tracking accuracy or failed attention checks, resulting in a final sample of 76 participants (positive condition: n=42; negative condition: n=34). An a priori power analysis (G*Power; α=.05; power=0.95; effect size=0.80) indicated a required sample size of at least 35 participants per group, confirming sufficient statistical power. In response to the reviewers’ and editor’s comments, we have streamlined and refined our hypotheses. Importantly, this revision involved a structural clarification rather than changes to the underlying analyses or hypothesis testing. The updated hypotheses and their correspondence to the original versions are presented in Table S1 in Multimedia Appendix 1.

### Eye-Tracking Setup

Gaze data (fixation duration) were recorded using a Tobii Pro Spark eye tracker (60 Hz), mounted below a 22-inch laptop screen (1920×1080 pixels, 60 Hz). Sessions were conducted individually in a controlled laboratory environment, with participants seated approximately 45‐55 cm from the screen [70]. A standard 9-point calibration was performed prior to the experiment, and only calibrations with a validation accuracy below 0.80° were accepted [71].

### Materials

The stimulus for the eye-tracking experiment was a mock-up of an Apple app store listing for a fictitious skin cancer screening app (“SkinScan”), modeled after existing apps. To reduce familiarity bias, app name and content were adapted. Two versions of SkinScan were created, identical in all aspects except for the middle of the 3 user star ratings: the positive rating condition showed two 5-star ratings and one 4-star rating, while the negative rating condition replaced the middle 5-star with a 1-star rating to introduce the experimental manipulation (Multimedia Appendix 2). All other elements, including the overall star rating and review text, were identical across conditions. Both app versions were integrated into and presented through Tobii Pro Lab.

### Procedure

**Figure 2.**
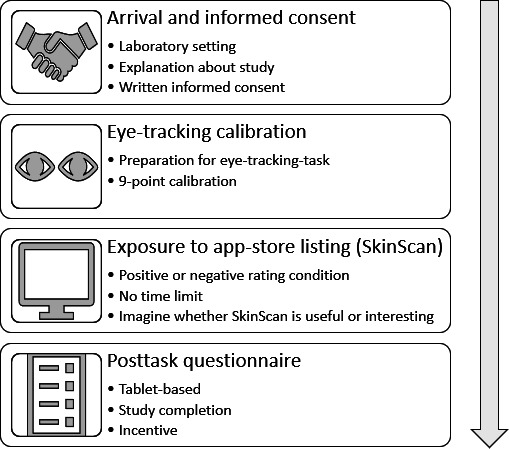
Schematic representation of the study workflow.

After providing informed consent, participants were seated in front of the eye-tracking system. They were instructed to view the app page as they normally would and to imagine evaluating its usefulness. Subsequently, a 9-point calibration procedure was conducted. Following calibration, the experiment began. No time limit was imposed, and gaze behavior was recorded continuously throughout the task. After stimulus exposure, participants completed a questionnaire on a tablet device and received compensation (see Figure 2 for the procedure of the experiment). A small pretest (n=3) using a think-aloud approach was conducted to ensure clarity and usability of the study design.

### Posttask Questionnaire

The questionnaire consisted of 29 items. The first section measured PEOU, PU, trust, and BI using validated multi-item scales, with each construct measured by 4 items (see Table S2 in Multimedia Appendix 1 for a complete list of items and scale specifications). The second section assessed the self-rated importance of app attributes (eg, price, reviews, and data protection) on 5-point Likert scales (1=not at all important and 5=very important). The third section included self-rated AI knowledge, WTP, and demographic variables (age, sex, previous experience with health apps, and education). All scales were adapted from prior research. Following established scale development procedures [72,73], content modifications were made to reflect the study’s objectives. To ensure linguistic accuracy, all items were translated using a back-translation procedure [74]. An attention-check item was included to ensure data quality. A full list of questionnaire items is provided in Table S2 in Multimedia Appendix 1.

### Data Analysis

To assess potential common method bias, the Harman single-factor test was conducted [75]. An exploratory factor analysis including all questionnaire items was performed. Parallel analysis indicated a multifactor structure, and the single-factor solution accounted for only 30% of the total variance. Moreover, the 1-factor model showed poor fit (*χ*²_135_=4364.2; *P*<.001). These results suggest that common method bias is unlikely to be a significant concern in this study.

Fixation durations (in seconds) were recorded within predefined AOIs (Multimedia Appendix 3) and processed using Tobii Pro Lab. Fixation duration data were aggregated into composite categories (eg, reviews, ratings, and description) and normalized by AOI size (seconds per square pixel) to account for differences in area. Mean fixation durations per AOI were then calculated at the participant level. Participants with insufficient tracking accuracy or missing data were excluded (n=19), yielding a final sample of 76.

Prior to analysis, scatterplots were examined to assess linearity and identify influential outliers. Pearson correlations were used to test relationships between variables, and independent-samples 2-tailed *t* tests (including Cohen *d*) were conducted to compare experimental conditions. Multiple regression analyses were performed to assess predictors of BI and WTP. Mediation and moderation effects were tested using regression-based approaches with bootstrapped CIs. All analyses were conducted at a significance level of *P*<.05, with 95% CIs reported.

### Ethical Considerations

The authors assert that all procedures contributing to this work comply with the ethical standards of the relevant national and institutional committees on human experimentation and with the Helsinki Declaration of 1975, as revised in 2008. An ethics approval was granted by the University of Applied Sciences and Arts Bielefeld (2025_002), and the study was preregistered. Informed consent was required from all participants in this study. Participants received approximately US $5.83 compensation.

## Results

### Sample Characteristics

The final sample consisted of 76 participants. Of these, 42 of 76 (55%) participants were randomly assigned to view the positive rating condition, while 34 of 76 (45%) participants saw the negative rating condition. As shown in Table 1, participants had a mean age of 22.88 (SD 3.13; range 18-33) years and a median age of 22 (IQR 21-24) years. The majority of participants were female (53/76, 70%). Most reported having a general school leaving certificate (49/76, 64%), while 13 of 76 (17%) held a university degree. The mean level of self-rated AI knowledge was 2.65 (SD 0.91). None of the participants had prior experience with skin cancer screening apps.

**Table 1.** Characteristics of the study sample.

Characteristics	Values
Age (years)	
Mean (SD)	22.88 (3.13)
Range	18-33
Median (IQR)	22 (21-24)
Sex, n (%)	
Female	53 (70)
Male	23 (30)
Education, n (%)	
General school leaving certificate (eg, Abitur)	49 (64)
Vocational school qualification (eg, apprenticeship)	12 (16)
University degree (eg, bachelor)	13 (17)
PhD	—^a^
Other	1 (1)
No answer	1 (1)
Previous experience with skin cancer screening apps, n (%)	
Yes	—^a^
No	76 (100)

a Not available.

### Effects of Rating Valence on Technology Acceptance

To address the research question of how a single negative rating affects behavioral intentions, independent-samples comparisons were conducted to examine the effect of rating valence on trust, PU, PEOU, BI, and WTP (Table 2). The results indicated no significant differences between positive and negative rating conditions for any of the examined variables (all *P*s>.05). Thus, H1a-e are not supported.

**Table 2.** Effects of rating valence on the technology acceptance model constructs.

Hypothesis (H)	Variable	Positive rating (n=42), mean (SD)	Negative rating (n=34), mean (SD)	*P* value, overall	Cohen *d*
H1a	Trust	2.48 (0.61)	2.62 (0.70)	.35	0.22
H1b	PU^a^	3.78 (0.64)	3.71 (0.71)	.64	0.11
H1c	PEOU^b^	4.23 (0.48)	4.12 (0.62)	.41	0.20
H1d	BI^c^	2.96 (0.84)	3.14 (1.01)	.42	−0.19
H1e	WTP^d^	19.5 (76.4)	63.1 (229)	.30	−0.27

a PU: perceived usefulness.

b PEOU: perceived ease of use.

c BI: behavioral intention to use.

d WTP: willingness to pay.

### Extending the TAM

**Figure 3.**
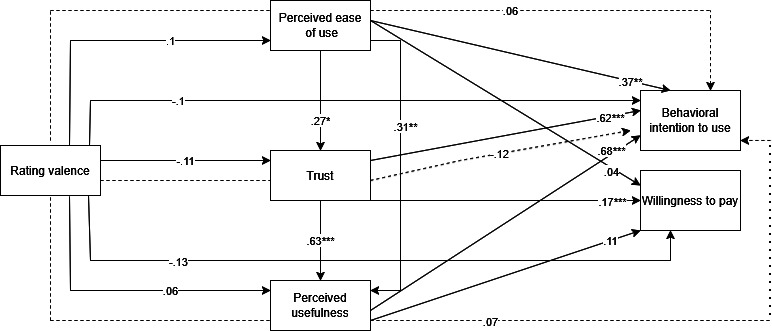
Summary of the results of the extended technology acceptance model. Dashed lines illustrate mediation analysis. H: hypothesis. *P=.05, **P<.01, ***P<.001.

To investigate the research question concerning the applicability of the TAM framework in connecting rating valence to behavioral intentions, Pearson correlation coefficients were calculated to examine the strength of the relationships between the hypothesized constructs (Figure 3). PEOU was positively correlated with PU (H2b; *r*=0.31; *P*=.007) and trust (H2a; *r*=0.27; *P*=.02). Trust also showed a strong positive correlation with PU (H2c; *r*=0.63; *P*<.001). Regarding BI, all proposed constructs were significantly positively correlated: PEOU (H3a; *r*=0.37; *P*=.001), trust (H3b; *r*=0.62; *P*<.001), and PU (H3c; *r*=0.68; *P*<.001). In addition, Pearson correlation analyses revealed a small but significant positive association between trust and WTP (H4b; *r*=0.17; *P*<.001). In contrast, WTP was not significantly correlated to PU (H4c; *r*=0.11; *P*=.35) or PEOU (H4a; *r*=0.04; *P*=.76).

To further examine the predictors of BI, a multiple linear regression analysis was conducted including rating valence (condition), PU, PEOU, and trust. The overall model was significant (*F*_4,71_=22.36; *P*<.001), explaining a substantial proportion of variance in behavioral intention (*R*^2^=0.56; adjusted *R*^2^=0.53). PU emerged as the strongest predictor of BI (β=.65; *P*<.001). Trust also showed a significant positive effect (β=.37; *P*=.01), while PEOU had a marginal positive influence (β=.27; *P*=.06). In contrast, rating valence (condition) did not significantly predict BI (β=−.20; *P*=.18). Overall, the findings closely resemble the individual correlations, with the exception that the effect of trust weakens once PU is included, pointing toward a potential mediation effect.

To examine whether rating valence and the TAM-related constructs predict WTP, a multiple linear regression analysis was conducted including condition, PU, PEOU, and trust. The overall model was not significant (*F*_4,71_=0.77; *P*=.55) and explained only a negligible proportion of variance in WTP (*R*^2^=0.04; adjusted *R*^2^=−0.01). None of the predictors showed a significant effect on WTP, including rating valence (β=−.20; *P*=.32), PU (β=.02; *P*=.85), PEOU (β=.00; *P*=.99), and trust (β=.10; *P*=.39). Overall, the regression results largely mirror the correlation findings. However, the initially observed association between trust and WTP did not remain significant in the regression model, suggesting that this relationship is weak and not robust when controlling for other variables.

To examine whether the effect of rating valence on BI is mediated by PEOU, PU, and trust (H5a-c), mediation analyses were conducted (see Table 3 and dashed lines in Figure 3). Results revealed no significant indirect effects for any of the mediators, as all CIs included 0. Additionally, no significant total or direct effects of rating valence were observed. Thus, H5 is not supported.

**Table 3.** Mediation effects of rating valence on behavioral intention to use via PEOU^a^, PU^b^, and trust.

Hypothesis (H)	Mediator	Indirect effect (ACME^c^), estimate (95% CI)	Direct effect (ADE^d^), estimate (95% CI)	Total effect, estimate (95% CI)
H5a	PEOU	0.06 (−0.10 to 0.25)	−0.24 (−0.63 to 0.18)	−0.17 (−0.57 to 0.25)
H5b	PU	0.07 (−0.22 to 0.35)	−0.24 (−0.56 to 0.05)	−0.17 (−0.60 to 0.23)
H5c	Trust	−0.12 (−0.40 to 0.14)	−0.05 (−0.38 to 0.30)	−0.17 (−0.55 to 0.28)

a PEOU: perceived ease of use.

b PU: perceived usefulness.

c ACME: average causal mediation effect.

d ADE: average direct effect.

### Visual Attention and the Processing of Heuristic Cues

To examine the research question of where users look for information when browsing for a skin cancer screening app, we analyzed how participants allocate visual attention across the app store interface. During data preprocessing, individual AOIs were aggregated into composite AOIs. Specifically, all review-related AOIs were combined into a reviews-AOI, all rating-related AOIs into a ratings-AOI, icon and screenshots into a design-AOI, and information and description into a description-AOI. To control for differences in AOI size, fixation durations were normalized and expressed as seconds per pixel² (s/px^2^). As shown in Table 4, the description-AOI attracted the longest fixation durations (mean 0.16, SD 0.10 s/px^2^), followed by the reviews-AOI (mean 0.11, SD 0.07 s/px^2^) and the ratings-AOI (mean 0.04, SD 0.04 s/px^2^). In contrast, the price-AOI (mean 0.02, SD 0.03 s/px^2^) and the data protection-AOI (mean 0.02, SD 0.02 s/px^2^) received the least attention.

**Table 4.** Normalized fixation durations by area of interest (AOI).

AOI	Values, n	Mean (SD) (s/px^2^)	95% CI
Design	76	0.04 (0.02)	0.04‐0.05
Description	76	0.16 (0.10)	0.14‐0.19
Reviews	76	0.11 (0.07)	0.09‐0.13
Ratings	68	0.04 (0.04)	0.03‐0.05
Data protection	76	0.02 (0.02)	0.02‐0.03
Price	76	0.02 (0.03)	0.01‐0.03

To examine the research question of whether the self-rated importance of app attributes is reflected in users’ gaze patterns, Pearson correlations were computed between participants’ ratings and normalized fixation durations on the corresponding AOIs (Table 5). Results revealed a significant positive correlation for reviews (*r*=0.28; *P*=.01), while all other relationships were nonsignificant (*P*s>.05). Thus, only H6b is supported.

**Table 5.** Pearson correlations between participants’ self-rated importance of app attributes and fixation durations on corresponding areas of interest (AOIs).

Hypothesis (H)	Normalized fixation durations on AOI (s/px^2^)	Self-rated importance of app attributes	App attribute, mean (SD)	*r* (95% CI)
H6a	Design	Importance of design	4.03 (0.83)	−0.11 (–0.32 to 0.11)
H6b	Reviews	Importance of reviews	4.21 (0.80)	0.28^a^ (0.05 to 0.47)
H6c	Ratings	Importance of reviews	4.21 (0.80)	0.17 (–0.07 to 0.39)
H6d	Price	Importance of price	4.57 (0.63)	≈0 (–0.26 to 0.22)
H6e	Description	Importance of description	4.72 (0.53)	−0.21 (–0.41 to 0.01)
H6f	Data protection	Importance of data protection	3.82 (1.05)	0.08 (–0.14 to 0.30)

a *P*<.05.

To address the research question of how rating valence affects gaze patterns, fixation duration on the manipulated review-AOI was compared across rating conditions. Absolute fixation durations were used, as the AOI size was identical across conditions. Results showed no significant difference between the positive (mean 6.17, SD 4.15 seconds) and negative conditions (mean 6.21, SD 3.98 seconds; *P*=.97; *d*=−0.01). Although participants in the negative condition showed a slight tendency toward longer viewing times, this effect was not statistically significant. Thus, H7 is not supported.

### Moderating Role of Attention and Individual Differences

To test whether visual attention moderates the relationship between rating valence and key constructs (H8a-d), moderation analyses were conducted using fixation durations on the ratings-AOI (Table S3 in Multimedia Appendix 1). Results indicated no significant interaction effects between rating valence and fixation durations on PU, PEOU, trust, or BI (all *P*s>.05). Thus, H8 is not supported.

Analyses of individual differences revealed no significant effects of demographic or experiential variables on the main outcome measures (trust, PU, PEOU, BI, and WTP; all *P*s>.05). Notably, both age and prior experience showed limited variance within the sample, which restricts the interpretability of their relationships with the outcome variables. In particular, none of the participants reported prior experience with skin cancer screening apps. Furthermore, self-reported AI knowledge was not significantly associated with any of the outcome variables (all *P*s>.05). Similarly, no significant differences were observed across sex or education levels (all *P*s>.05)

### Exploratory Analysis

Furthermore, we explored potential sex differences in self-rated app attribute importance and gaze patterns. Overall, female and male participants evaluated most app attributes similarly. No significant sex differences were observed for the importance of price, data protection, or evidence (all *P*s>.05). However, female participants rated the importance of reviews (*d*=0.63; *P*=.01) and design (*d*=0.69; *P*=.02) significantly higher than male participants (Table 6).

**Table 6.** Sex differences in self-rated importance of app attributes and normalized area of interest (AOI) fixation durations.

Measure and attribute or AOI		Female (n=53), mean (SD)	Male (n=23), mean (SD)	*P* value, overall	Cohen *d*
Self-rated importance					
Price		4.66 (0.52)	4.39 (0.84)	.17	0.43
Data protection		3.83 (1.03)	3.83 (1.11)	.99	<.001
Evidence		4.70 (0.57)	4.78 (0.42)	.48	−0.16
Reviews		2.36 (0.79)	3.87 (0.76)	.01^a^	0.63
Design		4.21 (0.72)	3.65 (0.98)	.02^a^	0.69
Normalized fixation durations on AOI (s/px^2^)					
Price		0.02 (0.03)	0.02 (0.03)	.81	−0.05
Data protection		0.03 (0.03)	0.02 (0.02)	.26	0.24
Ratings		0.05 (0.05)	0.03 (0.02)	.03^a^	0.47
Reviews		0.12 (0.07)	0.09 (0.05)	.12	0.34
Design		0.05 (0.03)	0.04 (0.01)	.08	0.36
Description		0.17 (0.11)	0.16 (0.07)	.46	0.15

a *P*<.05.

With regard to gaze patterns, female participants demonstrated longer fixation durations than male participants across all AOIs; however, this difference reached statistical significance only for ratings (*d*=0.47; *P*=.03). No significant sex differences were observed for fixation durations on data protection, description, reviews, design, or price (all *P*s>.05; Table 6).

## Discussion

### Principal Findings

This study investigated the acceptance of AISCSAs by integrating the TAM with eye-tracking and the experimental manipulation of rating valence. Importantly, by systematically varying rating valence as an external heuristic cue, the study enabled a direct test of how such cues causally influence the formation of PU, PEOU, and trust, as well as BI and WTP. Our findings reveal a hierarchy of visual attention dominated by textual descriptions and social proof while also identifying a significant gap between self-reported attribute importance and actual gaze behavior. Although the core TAM constructs (PU, PEOU, and trust) emerged as robust predictors of BI, the experimental manipulation of rating valence did not significantly alter acceptance beliefs or attention patterns in this specific laboratory context.

### Effects of Rating Valence on Acceptance and Attention

Contrary to our expectations, rating valence did not significantly influence trust, PU, PEOU, BI, or WTP. Likewise, the negative rating condition did not increase visual attention to the review section. Thus, neither H1 nor H7 was supported. At first glance, these findings appear inconsistent with research on negativity bias and online review effects, which often show that negative information attracts more attention and exerts a disproportionately strong influence on judgments [24,60]. However, our findings are more plausibly interpreted as evidence of a boundary condition than as evidence against the relevance of rating valence per se.

A first explanation concerns the subtlety of the manipulation. In this study, rating valence was altered only minimally, replacing 1 of 3 visible ratings with a single 1-star evaluation, while all other cues remained favorable. Such a minor discrepancy may have been insufficient to shift appraisals of a high-stakes health app. This is consistent with meta-analytic evidence by Purnawirawan et al [76], which demonstrates that valence effects are not uniform; rather, they are highly dependent on the degree of extremity and the evaluative context, such as product type and brand familiarity. In high-stakes environments, a single negative cue may lack the weight necessary to override a predominantly positive information environment. In addition, in high-stakes contexts, users may engage in more motivated and systematic processing, thereby reducing the relative impact of simple heuristic cues such as rating valence.

A second explanation is that app store ratings may have limited informational value in digital health contexts. Several studies show that consumer ratings are poor proxies for clinically relevant attributes such as quality, safety, and privacy. For example, Hyzy et al [77] found that ratings and downloads do not reliably reflect professional assessments, while de Chantal et al [78] reported a clear gap between user ratings and expert evaluations. Similarly, Levine et al [79] demonstrated that star ratings correlate only weakly with critical dimensions such as privacy, security, and interoperability, with even highly rated apps often performing poorly in these areas. This suggests that rating information may not provide users with meaningful signals for evaluating clinically relevant aspects of health apps. As a result, ratings may play a limited role in shaping acceptance beliefs.

From a dual-process perspective, the results indicate that the manipulated rating did not cross the threshold required to redirect processing from a default heuristic impression (type 1) to more effortful elaboration (type 2). While negative information can trigger deeper scrutiny [62], this is most likely when it is salient, coherent, and clearly diagnostic. Here, the cue may have been noticeable but not sufficiently consequential to reorganize attention or alter technology beliefs. This interpretation also fits the absence of any moderation by fixation durations on the ratings-AOI (H8): without a sufficiently strong evaluative signal, there was little for visual attention to amplify. Importantly, the null findings for H1 and H7 should not be interpreted as evidence that rating valence is irrelevant in digital health. Rather, they indicate that isolated, weakly negative rating cues are insufficient to meaningfully influence acceptance beliefs or increase visual attention in a high-risk medical AI context.

### TAM Validity and the Role of Trust in AI Health Apps

Despite the absence of direct effects of rating valence, the internal structure of the extended TAM was strongly supported. The positive relationships between PEOU, PU, and trust (H2a-c), as well as their associations with BI (H3a-c), are consistent with prior research, demonstrating the robustness of the TAM in digital health contexts [37,40,80]. The regression results further refine this picture by highlighting a differentiated role of the constructs: PU emerged as the strongest predictor of BI, followed by trust, while PEOU showed only a marginal effect. This pattern suggests that usefulness constitutes the primary decision criterion, whereas ease of use operates mainly indirectly via PU.

The clear hierarchy of predictors reveals a specific decision-making logic in high-stakes AI health contexts. The dominance of PU suggests that for potentially life-impacting technologies such as AISCSAs, users follow a strongly instrumental evaluation logic, prioritizing diagnostic utility over interface convenience. In such contexts, perceived functional value appears capable of compensating for usability barriers, indicating that effectiveness outweighs ease of use. This finding is consistent with prior research in health care contexts, showing that PU is typically the strongest predictor of behavioral intention [34,40].

At the same time, our findings highlight the important role of trust in AI-based health care. While PU emerged as the strongest predictor of BI, trust remains as an independent predictor and is particularly relevant in contexts characterized by uncertainty and limited user expertise. In such settings, users rely on systems whose diagnostic processes are not easily interpretable, making trust a necessary condition for acting on PU. In this sense, trust may function as an enabling mechanism or boundary condition that determines whether PU translates into behavioral intention. This interpretation aligns with trust theory, which emphasizes its importance under conditions of uncertainty and risk [81,82], and with prior extensions of the TAM that position trust as a core belief construct [34,83-86].

Interestingly, the marginal effect of PEOU challenges the traditional emphasis on usability as a key driver of technology acceptance [13] and aligns with prior research, showing that PU is typically the dominant predictor, particularly in high-stakes contexts [34,37]. In this study, PEOU appears to play a secondary role once a system is perceived as useful and trustworthy. Users may be willing to tolerate complexity if it is associated with higher perceived competence or clinical rigor. This suggests that simplicity is not the primary objective in AI-based health care apps, with reliability and effectiveness outweighing usability considerations.

In contrast, WTP was not explained by the included variables. As WTP was measured using an open-ended response format, the variable was not normally distributed. To account for this, additional nonparametric correlation analyses (Spearman ρ) were conducted, yielding the same pattern of results. WTP was not significantly associated with PU or PEOU, and its relationship with trust remained weak (H4a and H6c not supported). This indicates that WTP represents a distinct and less stable evaluative judgment that cannot be fully explained by the traditional TAM constructs [23,28]. Instead, monetary evaluations appear to be driven more strongly by psychological and normative factors, such as perceived risk, fairness, and emotional or social considerations [27]. Accordingly, while the TAM provides a robust framework for explaining acceptance intentions, it appears less suitable for capturing economic decision-making without incorporating additional constructs.

The lack of significant mediation (H5) and moderation (H8) effects is directly tied to the nonsignificant main effect of rating valence. Without an initial “push” from the experimental condition, the “amplification effect” of visual attention, where fixations serve to increase the weighting of attended information [65,66], had no baseline signal to amplify. These findings underscore the robustness of the TAM framework while highlighting that single-cue manipulations may be insufficient to shift these deeply interrelated constructs.

### Visual Attention Allocation and Information Processing

The eye-tracking results reveal a clear attentional hierarchy: users primarily focused on the app description, followed by reviews and ratings, while price and data protection received little attention. This pattern suggests that, in high-stakes contexts such as AI-based health apps, users prioritize information that helps them understand the app’s functionality and potential clinical value, indicating more deliberate and analytical processing (type 2). This finding is consistent with prior research showing that textual information plays a central role in app evaluation, despite developers often emphasizing visual design [54,87].

At the same time, the secondary attention given to reviews and ratings highlights that social proof remains relevant, particularly under uncertainty. These cues are easily interpretable and visually salient, making them effective heuristics for initial evaluation and risk reduction [19,21,60]. However, the fact that they received comparatively less attention than the description suggests that social proof complements rather than dominates decision-making in this context. Overall, the findings indicate that users integrate both analytical and heuristic processing, with greater weight placed on substantive, information-rich content.

In contrast, the data protection-AOI and the price-AOI received minimal attention. The limited focus on these elements is consistent with prior eye-tracking research, showing that users often overlook pricing information and security indicators in digital environments [58,88,89]. Several factors may explain this pattern. First, many health apps follow freemium models, where pricing information is either absent or only becomes relevant after installation. Second, although users frequently report privacy as a major concern [90,91], the complexity and legalistic nature of privacy policies may discourage active engagement. In addition, users may rely on implicit trust in platform-level regulation or assume a baseline level of data protection for health-related apps [92]. Finally, the low visual salience of these sections likely contributes to their neglect, as prior research shows that subtle privacy indicators are easily overlooked, whereas more prominent cues attract attention [56]. Taken together, these findings provide behavioral evidence for the well-documented “privacy paradox”: despite expressing concerns about data protection, users allocate little attention to related information during decision-making.

### The Discrepancy Between Self-Reports and Gaze Patterns

A central finding of this study is the limited alignment between self-reported importance and actual visual attention, with the exception of reviews (H6b). While participants who rated reviews as important also allocated more attention to them, no such relationship was observed for design, price, evidence, or data protection (H6a and H6c-f). This discrepancy suggests that self-reports and gaze data capture different aspects of the decision-making process. In line with prior research, eye-tracking reflects implicit attentional processes that are not accessible through retrospective self-reports [41].

From a dual-process perspective, this divergence can be interpreted as a misalignment between type 1 and type 2 processing [14]. Self-reported importance ratings primarily reflect deliberative, reflective evaluations (type 2), often shaped by normative expectations or socially desirable responses, particularly in sensitive contexts such as health care. In contrast, eye-tracking captures spontaneous, heuristic-driven allocation of attention during actual interaction (type 1). This gap is particularly pronounced for data protection. Although participants rated privacy as highly important, it received little visual attention during the task. This pattern is consistent with prior findings on the “privacy paradox,” where expressed concerns do not translate into corresponding behavior [57,58]. Taken together, these findings highlight that technology acceptance cannot be understood as a purely rational process, as suggested by traditional TAM frameworks. Instead, it reflects an interplay between reflective evaluations and implicit attentional processes. By capturing these behaviorally grounded mechanisms, eye-tracking provides a valuable complement to self-report measures and offers a more nuanced understanding of how users evaluate health-related AI systems.

### Individual Differences in AI App Evaluation

Our exploratory data suggest sex-specific patterns in app evaluation, with female participants assigning greater importance to reviews and design and showing longer fixation durations on ratings. This aligns with prior research, indicating that female participants tend to engage in more comprehensive information processing and pay closer attention to user-generated content when evaluating digital products [60,67,93]. Beyond sex, recent studies highlight the broader relevance of sociodemographic factors in shaping AI acceptance in health. For instance, Aras et al [68] show that younger individuals, female participants, and those with higher income levels are more likely to adopt AI in health care. Similarly, Méndez-Suárez et al [69] find that positive attitudes toward AI-based products are more prevalent among younger individuals and those with higher socioeconomic status. Complementing these findings, Kauttonen et al [94] demonstrate that individuals with moderate knowledge exhibit the highest acceptance of AI apps in health, whereas both low and high knowledge levels are associated with more cautious evaluations.

The absence of significant effects for AI knowledge and prior experience in this study likely reflects the relative homogeneity of the student sample, which limits variability in key characteristics. This highlights an important boundary condition of the findings. Future research should therefore examine more diverse populations, particularly older users and individuals with varying socioeconomic backgrounds and levels of digital literacy, to better capture how individual differences shape trust and acceptance in AI-based health care contexts.

### Limitations

This study, while offering valuable insights, is subject to several limitations that should be acknowledged. First, although the stimulus used in the eye-tracking experiment ensured a high degree of ecological validity by closely resembling an actual app store page, the experimental environment and the task itself remained artificial and hypothetical; for instance, participants did not autonomously decide to search for an AISCSA. This setting may not fully capture the complexity and dynamics of real-world app evaluation and decision-making. Moreover, the absence of real consequences, such as actually downloading or using the app, could have influenced participants’ engagement, attention patterns, and responses. Second, all participants were young university students with a narrow age range and no prior experience with skin cancer screening apps. This homogeneity limits the generalizability of the findings to other populations, especially older users or individuals with specific health concerns, who may differ in their levels of trust, digital literacy, and motivation. Therefore, the findings should be interpreted with caution, particularly when generalizing to more diverse or clinically relevant populations. Third, the internal consistency of some constructs, such as PU, PEOU, and trust, was relatively low, with Cronbach α values falling slightly below the conventional 0.70 threshold. While these levels are often considered acceptable in exploratory research within emerging fields, they suggest a moderate reliability, which should be taken into account when interpreting the strength of the observed relationships. Future research could benefit from further refining these scales to enhance their psychometric robustness in the context of AI-based health apps.

### Conclusions

Addressing a key gap in prior research, this study extends the TAM by experimentally manipulating rating valence as an external variable and examining its effects on trust and core acceptance constructs. While prior research has largely relied on observational data, this approach enables a direct test of how external information influences the TAM-related belief structures.

The findings confirm that PU, PEOU, and trust remain significant predictors of behavioral intention in the context of AISCSAs. However, rating valence showed no direct effect on user acceptance or visual attention. This suggests that in high-stakes health contexts, users are less influenced by simple social-proof cues and instead prioritize substantive, information-rich content.

The primary implication for developers and marketers of health-related AI tools is that relying solely on positive ratings to drive adoption may be an insufficient strategy. Instead, efforts should be directed toward providing transparent, evidence-based information, as the description-AOI received the longest fixation duration. Building acceptance through verifiable information appears to be more critical than leveraging simple social proof in this domain. Moreover, designing a more appealing data protection section may increase user attention, rendering them better-informed consumers. Importantly, the weak or nonexistent associations between fixation durations on specific AOIs and the self-rated importance of app attributes indicate that eye-tracking captures aspects of information processing that are not directly reflected in self-reported questionnaires. These findings demonstrate that combining self-report measures with behavior-based methods, such as eye-tracking, provides a more comprehensive understanding of user acceptance processes in health-related AI systems. This dual approach captures both reflective, self-reported evaluations (type 2) and implicit, attention-driven processes (type 1), which are not accessible through questionnaires alone.

## Supplementary material

10.2196/93489 Multimedia Appendix 1 Overview of preregistered hypotheses from the original submission and revised hypotheses reported in this paper. The table summarizes modifications made in response to reviewer and editor feedback during the peer-review process.

10.2196/93489 Multimedia Appendix 2 Visual representation of the experimental stimulus: positive (top) and negative (bottom) rating conditions. The manipulation focuses on the central review, where the star rating was varied while keeping the review text identical to isolate the effect of rating valence on user perception.

10.2196/93489 Multimedia Appendix 3 Predefined areas of interest for the simulated app-store interface. The colored regions represent the specific areas used to fixation duration.

## Abbreviations

AI: artificial intelligence
AISCSA: artificial intelligence–based skin cancer screening app
AOI: area of interest
BI: behavioral intention to use
PEOU: perceived ease of use
PU: perceived usefulness
TAM: technology acceptance model
WTP: willingness to pay

## References

[1] (2021). S3-leitlinie prävention von hautkrebs. AWMF.

[2] (2022). Melanoma: assessment and management. NICE.

[3] Ersser SJ, Effah A, Dyson J (2019). Effectiveness of interventions to support the early detection of skin cancer through skin self-examination: a systematic review and meta-analysis. Br J Dermatol.

[4] Kis A, Augustin M, Augustin J (2017). Regionale fachärztliche versorgung und demographischer wandel in Deutschland—szenarien zur dermatologischen versorgung im jahr 2035 [Regional dermatology workforce supply and demographic change in Germany: scenarios for dermatologic care in 2035]. J Dtsch Dermatol Ges.

[5] Avilés-Izquierdo JA, Molina-López I, Rodríguez-Lomba E, Marquez-Rodas I, Suarez-Fernandez R, Lazaro-Ochaita P (2016). Who detects melanoma? Impact of detection patterns on characteristics and prognosis of patients with melanoma. J Am Acad Dermatol.

[6] Brinker TJ, Hekler A, Enk AH (2019). Deep learning outperformed 136 of 157 dermatologists in a head-to-head dermoscopic melanoma image classification task. Eur J Cancer.

[7] Codella NCF, Nguyen QB, Pankanti S (2017). Deep learning ensembles for melanoma recognition in dermoscopy images. IBM J Res Dev.

[8] Gaube S, Biebl I, Engelmann MKM, Kleine AK, Lermer E (2024). Comparing preferences for skin cancer screening: AI-enabled app vs dermatologist. Soc Sci Med.

[9] Jagemann I, Wensing O, Stegemann M, Hirschfeld G (2024). Acceptance of medical artificial intelligence in skin cancer screening: choice-based conjoint survey. JMIR Form Res.

[10] Baldauf M, Fröehlich P, Endl R, Cauchard J, Löchtefeld M Trust me, I’m a doctor—user perceptions of AI-driven apps for mobile health diagnosis.

[11] Young AT, Amara D, Bhattacharya A, Wei ML (2021). Patient and general public attitudes towards clinical artificial intelligence: a mixed methods systematic review. Lancet Digit Health.

[12] Longoni C, Bonezzi A, Morewedge CK (2019). Resistance to medical artificial intelligence. J Consum Res.

[13] Davis FD (1989). Perceived usefulness, perceived ease of use, and user acceptance of information technology. MIS Q.

[14] Evans J, Stanovich KE (2013). Dual-process theories of higher cognition: advancing the debate. Perspect Psychol Sci.

[15] Bickart B, Schindler RM (2001). Internet forums as influential sources of consumer information. J Interact Mark.

[16] Park S, McCallister J (2023). The effects of social proof marketing tactics on nudging consumer purchase. J Stud Res.

[17] Abdul Talib YY, Mat Saat R (2017). Social proof in social media shopping: an experimental design research. SHS Web Conf.

[18] Qiu K, Zhang L (2024). How online reviews affect purchase intention: a meta-analysis across contextual and cultural factors. Data Inform Manag.

[19] Luan J, Shan W, Wang Y, Xiao J (2019). How easy-to-process information influences consumers over time: online review vs. brand popularity. Comput Human Behav.

[20] Sällberg H, Wang S, Numminen E (2023). The combinatory role of online ratings and reviews in mobile app downloads: an empirical investigation of gaming and productivity apps from their initial app store launch. J Market Anal.

[21] Sung E, Chung WY, Lee D (2023). Factors that affect consumer trust in product quality: a focus on online reviews and shopping platforms. Humanit Soc Sci Commun.

[22] Kim Y, Peterson RA (2017). A meta-analysis of online trust relationships in e-commerce. J Interact Mark.

[23] Lee J, Oh Y, Kim M, Cho B, Shin J (2023). Willingness to use and pay for digital health care services according to 4 scenarios: results from a national survey. JMIR Mhealth Uhealth.

[24] Cui G, Lui HK, Guo X (2012). The effect of online consumer reviews on new product sales. Int J Electron Commer.

[25] Zhuang M, Cui G, Peng L (2018). Manufactured opinions: the effect of manipulating online product reviews. J Bus Res.

[26] Park C, Lee TM (2009). Information direction, website reputation and eWOM effect: a moderating role of product type. J Bus Res.

[27] Fu H, He W, Feng K, Guo X, Hou C (2024). Understanding consumers’ willingness to pay for circular products: a multiple model-comparison approach. Sustain Product Consum.

[28] Xie Z, Chen J, Or CK (2022). Consumers’ willingness to pay for eHealth and its influencing factors: systematic review and meta-analysis. J Med Internet Res.

[29] McDougall JA, Furnback WE, Wang BCM, Mahlich J (2020). Understanding the global measurement of willingness to pay in health. J Mark Access Health Policy.

[30] Lee AT, Ramasamy RK, Subbarao A (2025). Understanding psychosocial barriers to healthcare technology adoption: a review of TAM technology acceptance model and unified theory of acceptance and use of technology and UTAUT frameworks. Healthcare (Basel).

[31] Gulati S, Sousa S, Lamas D Modelling trust in human-like technologies.

[32] Sousa S, Lamas D, Dias P, Hutchison D, Kanade T, Kittler J (2014). Learning and Collaboration Technologies Designing and Developing Novel Learning Experiences.

[33] Kamal SA, Shafiq M, Kakria P (2020). Investigating acceptance of telemedicine services through an extended technology acceptance model (TAM). Technol Soc.

[34] Liu K, Tao D (2022). The roles of trust, personalization, loss of privacy, and anthropomorphism in public acceptance of smart healthcare services. Comput Human Behav.

[35] Matar A, Aloqaily AN (2025). The mediating influence of perceived usefulness on consumer behaviour towards driving e-wallet adoption in Jordan. J Open Innov.

[36] Pitts G, Motamedi S (2026). What drives students’ use of AI chatbots? Technology acceptance in conversational AI. arXiv.

[37] Adnan A, Irvine RE, Williams A, Harris M, Antonacci G (2025). Improving acceptability of mHealth apps-the use of the technology acceptance model to assess the acceptability of mHealth apps: systematic review. J Med Internet Res.

[38] Beldad AD, Hegner SM (2022). Running frequently with an app to be fantastic! Determinants of runtastic usage continuation intention among German users. Int J Mob Commun.

[39] Dhagarra D, Goswami M, Kumar G (2020). Impact of trust and privacy concerns on technology acceptance in healthcare: an Indian perspective. Int J Med Inform.

[40] Park JH, Lee CW, Do C (2025). Examining users’ acceptance intention of health applications based on the technology acceptance model. Healthcare (Basel).

[41] Nisbett RE, Wilson TD (1977). Telling more than we can know: verbal reports on mental processes. Psychol Rev.

[42] Matukin M, Ohme R, Boshoff C (2016). Toward a better understanding of advertising stimuli processing. J Advert Res.

[43] Segijn CM, Voorveld HAM, Vandeberg L, Smit EG (2017). The battle of the screens: unraveling attention allocation and memory effects when multiscreening. Hum Commun Res.

[44] Just MA, Carpenter PA (1980). A theory of reading: from eye fixations to comprehension. Psychol Rev.

[45] Rayner K (1998). Eye movements in reading and information processing: 20 years of research. Psychol Bull.

[46] Fu H, Manogaran G, Wu K, Cao M, Jiang S, Yang A (2020). Intelligent decision-making of online shopping behavior based on internet of things. Int J Inf Manage.

[47] Moravec PL, Minas RK, Dennis AR (2019). Fake news on social media: people believe what they want to believe when it makes no sense at all1. MIS Q.

[48] van der Gijp A, Ravesloot CJ, Jarodzka H (2017). How visual search relates to visual diagnostic performance: a narrative systematic review of eye-tracking research in radiology. Adv Health Sci Educ Theory Pract.

[49] Leiva LA, Xue Y, Bansal A Understanding visual saliency in mobile user interfaces.

[50] Li T, Pavlou PA (2014). How does social influence really affect consumer decisions? Insights from an eye tracking study. SSRN J.

[51] Maslowska E, Segijn CM, Vakeel KA, Viswanathan V (2020). How consumers attend to online reviews: an eye-tracking and network analysis approach. Int J Advert.

[52] Shan Y, Ji M, Xie W, Lam KY, Chow CY (2022). Public trust in artificial intelligence applications in mental health care: topic modeling analysis. JMIR Hum Factors.

[53] Shan W, Wang Y, Luan J, Tang P (2019). The influence of physician information on patients’ choice of physician in mHealth services using China’s Chunyu Doctor app: eye-tracking and questionnaire study. JMIR Mhealth Uhealth.

[54] Montazami A, Ann Pearson H, Kenneth Dubé A, Kacmaz G, Wen R, Shajeen Alam S (2022). Why this app? How educators choose a good educational app. Comput Educ.

[55] Ponnusamy M, Divya Venkatesh J, Das S, Nanda G (2025). HCI International 2025 Posters.

[56] Guerra M, Milanese R, Deodato M, Perozzi V, Fasano F Visual attention and privacy indicators in android: insights from eye tracking.

[57] Furman S, Theofanos M, Hutchison D, Kanade T, Kittler J (2014). Universal Access in Human-Computer Interaction: Design for All and Accessibility Practice.

[58] Pan Y, Ruan Y, Chang M, Lyu D, Li Y (2024). Read or skip privacy policies when installing apps on wearable devices: the roles of perceived necessity and threat clues. Humanit Soc Sci Commun.

[59] Duchowski A (2007). Eye Tracking Methodology.

[60] Chen T, Samaranayake P, Cen X, Qi M, Lan YC (2022). The impact of online reviews on consumers’ purchasing decisions: evidence from an eye-tracking study. Front Psychol.

[61] Skaramagkas V, Giannakakis G, Ktistakis E (2023). Review of eye tracking metrics involved in emotional and cognitive processes. IEEE Rev Biomed Eng.

[62] Fu H, Li Y, Wu Z, Tan Y, He W, Zuo J (2025). Immunization effect of inoculation messages: interventions against consensus and misinformation to improve public acceptance of recycled water. Humanit Soc Sci Commun.

[63] Chaiken S (1980). Heuristic versus systematic information processing and the use of source versus message cues in persuasion. J Pers Soc Psychol.

[64] Wedel M, Pieters R (2008). Eye tracking for visual marketing. Found Trends Mark.

[65] Armel KC, Beaumel A, Rangel A (2008). Biasing simple choices by manipulating relative visual attention. Judgm Decis Mak.

[66] Krajbich I, Armel C, Rangel A (2010). Visual fixations and the computation and comparison of value in simple choice. Nat Neurosci.

[67] Jagemann I, Stegemann M, von Brachel R, Hirschfeld G (2024). Gender differences in preferences for mental health apps in the general population—a choice-based conjoint analysis from Germany. BMC Psychiatry.

[68] Aras S, Drakos C, Manimangalam V (2025). Influencing public acceptance of artificial intelligence (AI) in healthcare delivery. Front Digit Health.

[69] Méndez-Suárez M, Monfort A, Hervas-Oliver JL (2023). Are you adopting artificial intelligence products? Social-demographic factors to explain customer acceptance. Eur Res Manag Bus Econ.

[70] (2023). How to position participants and the eye tracker. Tobii Pro.

[71] (2024). Tobii Pro Spark: product description. Tobii.

[72] Gerbing DW, Anderson JC (1988). An updated paradigm for scale development incorporating unidimensionality and its assessment. J Mark Res.

[73] Rossiter JR (2002). The C-OAR-SE procedure for scale development in marketing. Int J Res Mark.

[74] Brislin RW (1970). Back-translation for cross-cultural research. J Cross Cult Psychol.

[75] Fuller CM, Simmering MJ, Atinc G, Atinc Y, Babin BJ (2016). Common methods variance detection in business research. J Bus Res.

[76] Purnawirawan N, Eisend M, De Pelsmacker P, Dens N (2015). A meta-analytic investigation of the role of valence in online reviews. J Interact Mark.

[77] Hyzy M, Bond R, Mulvenna M (2024). Don’t judge a book or health app by its cover: user ratings and downloads are not linked to quality. PLoS One.

[78] de Chantal PL, Chagnon A, Cardinal M, Faieta J, Guertin A (2022). Evidence of user-expert gaps in health app ratings and implications for practice. Front Digit Health.

[79] Levine DM, Co Z, Newmark LP (2020). Design and testing of a mobile health application rating tool. NPJ Digit Med.

[80] Zin K, Kim S, Kim HS, Feyissa IF (2023). A study on technology acceptance of digital healthcare among older Korean adults using extended TAM (extended technology acceptance model). Adm Sci.

[81] Rousseau DM, Sitkin SB, Burt RS, Camerer C (1998). Not so different after all: a cross-discipline view of trust. Acad Manage Rev.

[82] Mayer RC, Davis JH, Schoorman FD (1995). An integrative model of organizational trust. Acad Manage Rev.

[83] Beldad AD, Hegner SM (2018). Expanding the technology acceptance model with the inclusion of trust, social influence, and health valuation to determine the predictors of German users’ willingness to continue using a fitness app: a structural equation modeling approach. Int J Hum Comput Interact.

[84] Benbasat I, Wang W (2005). Trust in and adoption of online recommendation agents. J Assoc Inform Syst.

[85] Hegner SM, Beldad AD, Brunswick GJ (2019). In automatic we trust: investigating the impact of trust, control, personality characteristics, and extrinsic and intrinsic motivations on the acceptance of autonomous vehicles. Int J Hum Comput Interact.

[86] Zhang T, Tao D, Qu X (2020). Automated vehicle acceptance in China: social influence and initial trust are key determinants. Transp Res Part C Emerg Technol.

[87] Kim E, Lin JS, Sung Y (2013). To app or not to app: engaging consumers via branded mobile apps. J Interact Advert.

[88] Arianezhad M, Camp LJ, Kelley T, Stebila D (2013). CODASPY ’13: Proceedings of the Third ACM Conference on Data and Application Security and Privacy.

[89] Whalen K, Inkpen M (2005). GI ’05: Proceedings of Graphics Interface 2005.

[90] Alhammad N, Alajlani M, Abd-Alrazaq A, Epiphaniou G, Arvanitis T (2024). Patients’ perspectives on the data confidentiality, privacy, and security of mHealth apps: systematic review. J Med Internet Res.

[91] Auxier B, Rainie L, Anderson M, Perrin A, Kumar M, Turner E (2019). Americans’ attitudes and experiences with privacy policies and laws. Pew Research Center.

[92] (2024). DiGA und DiPA datenschutzkriterien. Bundesinstitut für Arzneimittel und Medizinprodukte.

[93] Hwang YM, Lee KC (2018). Using an eye-tracking approach to explore gender differences in visual attention and shopping attitudes in an online shopping environment. Int J Hum Comput Interact.

[94] Kauttonen J, Rousi R, Alamäki A (2025). Trust and acceptance challenges in the adoption of AI applications in health care: quantitative survey analysis. J Med Internet Res.

[95] Jagemann I, Hegner S, Hirschfeld G (2026). Dataset for: augmenting self-reports: using eye-tracking and questionnaire data to elucidate the role of rating valence in AI skin cancer app acceptance. Zenodo.

