# Supplementary material for: The Role of Rating Valence in AI Skin Cancer App Acceptance: Eye-Tracking and Questionnaire Study
Source: JMIR Hum Factors. 2026 Jun 11;13:e93489. doi: 10.2196/93489 (PMC13258064; doi:10.2196/93489)
Supplement: Multimedia Appendix 1 [file humanfactors-v13-e93489-s001.docx]

# Supplementary material

Table S1. Overview of Original and Revised Hypotheses

| **Preregistered Hypothesis** | **Revised Hypothesis** |
| --- | --- |
| **H1 a-f:** The self-rated importances of app-attributes are correlated with fixation duration on the respective areas of interest (AOIs).  H1a): Visual design with Logo and Screenshot-AOI  H1b) Reviews with Review-AOI  H1c) Ratings with Rating-AOI  H1d) Price with Price-AOI  H1e) Evidence with Evidence-AOI  H1f) Data protection with Data protection-AOI | **H6:** There is a positive relationship between the self-rated importance of app attributes and actual visual attention (fixation duration) to the corresponding Areas of Interest (AOIs). This applies to: (a) visual design/Design-AOI, (b) user reviews/Review-AOI, (c) star ratings/Rating-AOI, (d) price/Price-AOI, (e) scientific evidence/Description-AOI, and (f) data protection/Data protection-AOI. |
| **H2:** Fixation duration on data protection-AOI is correlated with trust | Deleted |
| **H3**: Fixation duration on description-AOI is correlated with PU | Deleted |
| **H4**: Fixation duration on review-AOI is correlated with trust | Deleted |
| **H5:** Negative ratings increase attention to comments | **H7:** Negative ratings increase visual attention to user comments, reflected in longer fixation durations on the comments AOI. |
| **H6a-b**: Negative Ratings have a negative effect on a) Behavioral **Intention to Use** and b) **Willingness to Pay** | **H1:** Rating valence significantly influences user perceptions and intentions, such that negative ratings lead to lower (a) Trust, (b) PU, (c) PEOU, (d) BI, and (e) WTP. |
| **H7a-c:** Negative ratings negatively affect assumed mediators: a) **Trust, b) PU, c) PEOU** | **H1:** Rating valence significantly influences user perceptions and intentions, such that negative ratings lead to lower (a) Trust, (b) PU, (c) PEOU, (d) BI, and (e) WTP. |
| **H8:** PEOU is positively correlated with PU | **H2:** Within the AISCSA context, PEOU is positively associated with (a) Trust and (b) PU, and (c) Trust is positively associated with PU. |
| **H9**: Trust is positively correlated with PU | **H2**: Within the AISCSA context, PEOU is positively associated with (a) Trust and (b) PU, and (c) Trust is positively associated with PU. |
| **H10**: PEOU is positively correlated with Trust | **H2:** Within the AISCSA context, PEOU is positively associated with (a) Trust and (b) PU, and (c) Trust is positively associated with PU. |
| **H11:** The assumed mediators are positively correlated with Behavioural Intentions  H11a: PEOU is positively correlated with BI  H11b: PU is positively correlated with BI  H11c: Trust is positively correlated with BI | **H3:** BI is positively **associated with** (a) PEOU, (b) Trust, and (c) PU.  **H4:** WTP is positively **associated with** (a) PEOU, (b) Trust, and (c) PU. |
| **H12:** The assumed mediators mediate the effect of negative ratings on BI  H12a: PEOU mediates the effect of negative ratings on BI  H12b: PU mediates the effect of negative ratings on BI  H12c: Trust mediates the effect of negative ratings on BI | **H5:** The effect of rating valence on **Behavioral Intention** is mediated by **PEOU** (a), **PU** (b), and **Trust** (c). |
| **H13:** Fixation Duration on Rating-AOI moderate the effect of Ratings on a) PU, b) PEOU, c) Trust, d) BI | **H8:** The effect of rating valence on a) **PU**, b) **PEOU**, c) **Trust**, and d) **Behavioral Intention** is moderated by visual attention, such that higher fixation duration on the ratings AOI strengthens this relationship. |
| **H14-H18:** Additionally, we investigate how other predictors (age, experience, AI knowledge, gender, education) influence user perceptions and behavioral intentions. | Exploratory |

Table S2. Post-task questionnaire

| Construct  Reference | English Version | German Translation | Cronbachs Alpha |
| --- | --- | --- | --- |
| Perceived Usefulness  Inspired by:  Horsham et al. (2019),  α = .83 | PU1: I think SkinScan would help me examine a suspicious mole more quickly.  PU2: I think SkinScan could improve my ability to check my moles on my own.  PU3: I think that using SkinScan could contribute to the early detection of skin cancer.  PU4: I think that I would find SkinScan useful. | PU1: Ich denke, dass SkinScan mir helfen würde, einen verdächtigen Leberfleck schneller zu untersuchen.  PU2: Ich denke, dass SkinScan meine Fähigkeit verbessern könnte, meine Leberflecke eigenständig zu überprüfen.  PU3: Ich denke, dass die Nutzung von SkinScan zur frühzeitigen Erkennung von Hautkrebs beitragen könnte.  PU4: Ich denke, dass ich SkinScan nützlich finden würde. | .69 |
| Perceived Ease of Use  Inspired by:  Horsham et al. (2019),  α = .80 | PEOU1: I think that SkinScan would be clear and easy to understand.  PEOU2: I think that SkinScan would be easy for me to use.  PEOU3: I think installing SkinScan would be quick and easy.  PEOU4: I think SkinScan would provide an understandable diagnosis of a suspicious mole. | PEOU:1: Ich denke, dass die Nutzung von SkinScan klar und verständlich sein würde.  PEOU2: Ich denke, dass die Bedienung von SkinScan für mich einfach sein würde.  PEOU3: Ich denke, dass die Installation von SkinScan schnell und problemlos erfolgen würde.  PEOU4: Ich denke, dass die Diagnose eines verdächtigen Leberflecks durch SkinScan verständlich vermittelt werden würde. | .67 |
| Trust  Inspired by:  Hegner et al. (2019),  α = .87 | TRU1: I would have complete confidence in SkinScan.  TRU2: I would feel safe using SkinScan.  TRU3: I would rely on SkinScan's diagnosis of a suspicious mole.  TRU4: I would feel reassured if SkinScan indicated that my suspicious mole was normal. | TRU1: Ich hätte vollstes Vertrauen in SkinScan.  TRU2: Ich würde mich bei der Nutzung von SkinScan sicher fühlen.  TRU3: Ich würde mich bei einem verdächtigen Muttermal auf die Diagnose von SkinScan verlassen.  TRU4: Es würde mich beruhigen, wenn SkinScan anzeigt, dass mein verdächtiger Leberfleck unauffällig ist. | .68 |
| Behavioral Intention  Inspired by:  Horsham et al. (2019),  α = .84 | BI1: I intend to use SkinScan routinely in the future.  BI2: I expect to use SkinScan when I notice a suspicious mole.  BI3: I plan to use SkinScan whenever I want to get a diagnosis for a suspicious mole.  BI4: If I need an app for skin cancer detection, I would choose SkinScan. | BI1: Ich beabsichtige, SkinScan in Zukunft routinemäßig zu verwenden.  BI2: Ich gehe davon aus, SkinScan zu verwenden, wenn ich einen verdächtigen Leberfleck bemerke.  BI3: Ich plane, SkinScan zu nutzen, wann immer ich eine Diagnose für einen verdächtigen Leberfleck erhalten möchte.  BI4: Wenn ich eine App zur Hautkrebs-erkennung benötige, würde ich mich für SkinScan entscheiden. | .85 |
| Willingness to Pay | How much would you be willing to pay for a one-time diagnosis of a suspicious mole using SkinScan? | Wie viel wären Sie bereit, für eine einmalige Diagnose eines verdächtigen Leberflecks mit SkinScan zu bezahlen? |  |
| Self-reported AI knowledge | AI1: I am very knowledgeable about artificial intelligence (AI).  AI2: Compared to others, I consider my knowledge of artificial intelligence (AI) to be above average. | AI1: Ich kenne mich sehr gut mit Künstlicher Intelligenz (KI) aus.  AI2: Im Vergleich zu anderen schätze ich mein Wissen über Künstliche Intelligenz (KI) als überdurchschnittlich hoch ein. | .79 |
| Visual Design | How important is the visual design of apps to you? | Wie wichtig ist Ihnen das visuelle Design von Apps? |  |
| Price | How important is the price of apps to you? | Wie wichtig ist Ihnen der Preis von Apps? |  |
| Ratings & Reviews | How important are the reviews of other users of the app to you? | Wie wichtig sind Ihnen die Bewertungen anderer Nutzer der App? |  |
| Data-protection | How important is the app's data protection to you? | Wie wichtig ist Ihnen der Datenschutz der App? |  |
| Evidence | How important is it to you that the app is scientifically proven or evidence-based? | Wie wichtig ist Ihnen, dass die App wissenschaftlich belegt bzw. evidenzbasiert ist? |  |
| Age | How old are you? | Wie alt sind Sie? |  |
| Gender | Which gender do you identify with? | Welchem Geschlecht fühlen Sie sich zugehörig? |  |
| Education | What is the highest level of education you have attained? | Was ist Ihr höchster erreichter Bildungsabschluss? |  |
| Experience | Have you ever used an app to detect skin cancer? | Haben Sie schon einmal eine App zur Hautkrebs-Erkennung genutzt? |  |

Table S3. Fixation duration as a moderator between rating valence and model constructs

| **Hypothesis** | **Dependent**  **Variable** | **Rating Valence**  **ß (SE)** | **Ratings-AOI**  **ß (SE)** | **Interaction**  **ß (SE)** |
| --- | --- | --- | --- | --- |
| H8a | PU | -0.06 (0.23) | -0.00 (0.00) | 0.00 (0.00) |
| H8b | PEOU | 0.08 (0.20) | 0.00 (0.00) | 0.00 (0.00) |
| H8c | Trust | -0.05 (0.23) | 0.00 (0.00) | -0.00 (0.00) |
| H8d | BI | -0.53 (0.32) | -0.00 (0.00) | 0.00 (0.00) |
