# Supplementary figures and images for: The Role of Rating Valence in AI Skin Cancer App Acceptance: Eye-Tracking and Questionnaire Study
Source: JMIR Hum Factors. 2026 Jun 11;13:e93489. doi: 10.2196/93489 (PMC13258064; doi:10.2196/93489)

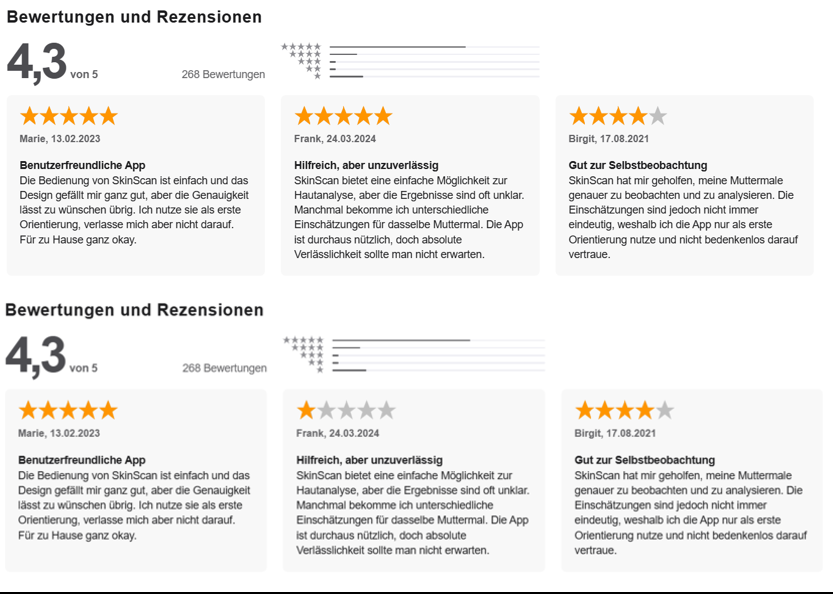

Supplement: Multimedia Appendix 2 [file humanfactors-v13-e93489-s002.png]

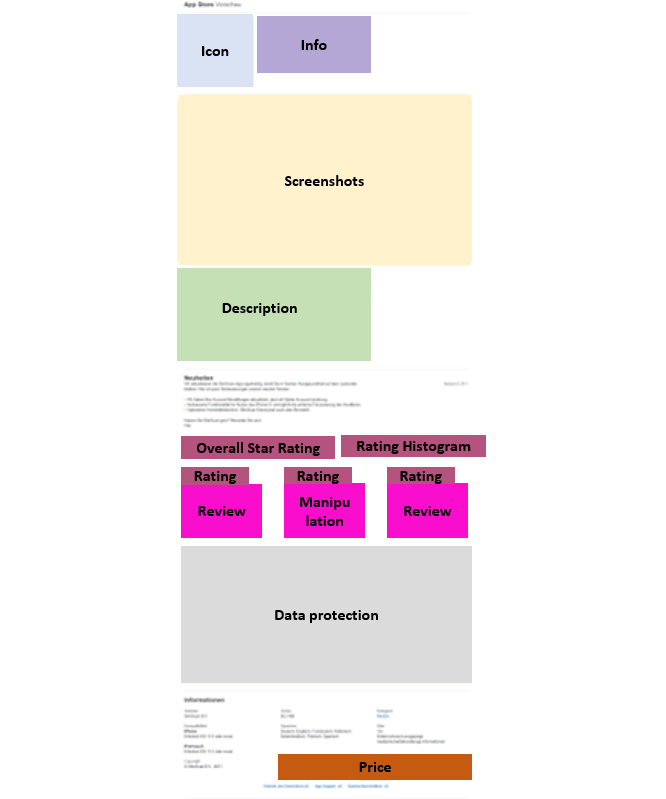

Supplement: Multimedia Appendix 3 [file humanfactors-v13-e93489-s003.png]
